# Contextual factors influencing the roles of patent medicine vendors in the provision of injectable contraception services in Nigeria

**DOI:** 10.1186/s12978-023-01650-8

**Published:** 2023-07-22

**Authors:** Ayodeji Matthew Adebayo, Mojisola Morenike Oluwasanu, Faizah Tosin Okunade, Olayinka Olufunke Ajayi, Akinwumi Oyewole Akindele, Ademola Johnson Ajuwon

**Affiliations:** 1 Department of Community Medicine, Faculty of Clinical Sciences, College of Medicine, University of Ibadan, Ibadan, Nigeria; 2 Department of Health Promotion and Education, Faculty of Public Health, College of Medicine, University of Ibadan, Ibadan, Nigeria; 3 Sightsavers International, Kaduna, Nigeria; 4 Department of Community Medicine, University College Hospital, Ibadan, Nigeria

**Keywords:** Contextual factors, Injectable contraceptives, Proprietary patent medicine vendors, Family planning, Access to healthcare

## Abstract

**Background:**

Patent medicine vendors (PMVs) play vital roles in the delivery of family planning services in Nigeria and other developing countries. There is a growing recognition of the need to integrate them into the formal health care system as a strategy to increase the contraceptive prevalence rate and achieve universal health coverage. Though promising, the success of this proposition is largely dependent on a critical analysis of the factors which influence their operations. This study was designed to identify the contextual factors influencing the provision of injectable contraceptive services by PMVs and the broader effects of their activities on the health system to inform similar interventions in Nigeria.

**Methods:**

This was a qualitative study guided by the UK Medical Research Council’s Framework for Complex Interventions. Twenty-seven in-depth interviews were conducted among officials of the association of PMVs, health workers, government regulatory officers and programme implementers who participated in a phased 3-year (2015–2018) intervention designed to enhance the capacity of PMVs to deliver injectable contraceptive services. The data were transcribed and analyzed thematically using NVIVO software.

**Results:**

The contextual factors which had implications on the roles of PMVs were socio-cultural and religious, the failing Nigerian health system coupled with government regulatory policies. Other factors were interprofessional tensions and rivalry between the PMVs and some categories of health care workers and increasing donors’ interest in exploring the potentials of PMVs for expanded healthcare service provision. According to the respondents, the PMVs bridged the Nigerian health system service delivery gaps serving as the first point of contact for injectable contraceptive services and this increased contraceptive uptake in the study sites. A negative effect of their operation is the tendency to exceed their service provision limits, which has spurred a planned tiered PMV accreditation system.

**Conclusions:**

This study has highlighted the contextual factors which define the roles and scope of practice of PMVs involved in injectable contraceptive service provision. Strategies and interventions aimed at expanding the healthcare delivery roles of PMVs must be encompassing to address the broader contextual factors which underpin their capacities and functions.

## Contributions to the literature

- This study highlighted the important roles of patent medicine vendors in injectable service delivery.
- This study outlined contextual factors with implications on why and the extent to which patent medicine vendors practised injectable contraceptive service provision.
- The study showed that the legal and regulatory environment largely influenced the scope of patent medicine vendors’ practice of injectable service delivery and underpins their capacities and functions.
- The study also revealed that deficiency in the formal health system which is unable to meet the health needs of the citizenry made it possible for patent medicine vendors to respond to the injectable contraceptive needs of women.

## Introduction

In Nigeria and other low- and middle-income countries (LMICs), Patent Medicine Vendors (PMVs) perform essential roles in the provision of health care including family planning services [1–3]. Nigeria has a growing population coupled with inadequate numbers of trained health care providers to cater for the healthcare needs of the populace. Also, the few available health care providers are not willing to work and live in the rural areas, thus, leaving a gap in human resource for health that is being readily filled by the PMVs.

Patent medicine vendor (PMV) drug shops are located in close proximity to the communities, and are frequently the first point of call for health information, medications, treatment for childhood illnesses, and family planning commodities particularly in neighbourhoods with few health facilities, pharmacies and community health workers [2]. Drug shops operated by PMVs are also more conspicuous than the family planning clinics and offer injectables making them the desirable choice by clients for their contraceptive needs [1]. Given their ubiquitous nature as well as their market share and accessibility, PMV drug shops are potential points of primary health care services in Nigeria [1–3]. Injectable contraceptives given outside the formal health care system have been found to be safe if the providers are trained and supervised adequately [4]. In the past, some vertical programs have confirmed that PMVs can be trained to deliver some level of healthcare interventions in the communities including treatment of diarrhea, pneumonia, and malaria [5]. The delivery of these vertical programs among PMVs were done in conjunction with relevant stakeholders such as the Pharmaceutical Council of Nigeria (PCN) and National Association of Patent Medicine Vendors (NAPPMED) [5].

In 2014, the Government of Nigeria launched a task-shifting and task-sharing policy for essential healthcare services [6] to address shortages in the availability of health workers needed to deliver essential health services. The policy called for an increase in the capacity of people in the community, including drug vendors, to provide treatment, counselling, and referral for some reproductive and maternal and child health services, including malaria treatment [6]. Taking advantage of the policy on task shifting, a consortium of stakeholders namely, the Population Council, Federal Ministry of Health (FMOH), State Ministries of Health (SMOH) and NAPPMED implemented a two-phased implementation research that trained some PMVs to deliver injectable contraceptive services in drug shops in Nigeria between 2015 and 2018. Phase one of the project was carried out in Oyo and Nasarawa States, both in South-West and North-Central regions respectively, between November 2015 and December 2016 [4]. The second phase was conducted in four states of the remaining four regions of North-East (Bauchi), North-West (Kaduna), South-East (Ebonyi) and South-South (Cross Rivers) between May 2017 and September 2018 [7]. A total of 381 PMVs participated in the two phases with 156 participants selected from 4 (2 urban and 2 rural) Local Government Areas (LGAs) in phase one [4, 7] and 225 recruited in phase two [7].

The PMVs attended a five-day training on family planning counselling, sale, referral, and administration of progestin-only injectable contraceptives. The training modules consisted of screening and counselling clients who are interested in the injectables; intramuscular and subcutaneous injectable contraceptives and their administration; safe storage and proper disposal of sharps; reinjection procedures, record keeping, procedures for handling adverse events; and referral [4, 7]. The intervention entailed the monitoring of the activities of trained PMVs as well as interviewing of clients who got injectable contraceptives at the third, sixth and ninth months in-order to determine factors affecting the continuation or discontinuation of use of injectables from the same PMVs or others. After completion of the training, the PMVs were required to offer services such as counselling, injecting, selling and referring clients who needed injectable contraceptives. The training expectations involved keeping complete records of socio-demographics including age, type of injectable service provided as well as telephone number of all clients who receive injectable contraceptives in their shops. The program was adjudged effective because few PMVs had the necessary knowledge to provide injectables prior to the training but a majority demonstrated increased knowledge after the training [4]. In addition, evaluation of the intervention showed that 83% of trained PMVs had provided injectable contraceptives to at least one client 30 days prior to the survey [4].

Although this intervention was effective in improving healthcare practice [8, 9] the contextual factors that influenced the provision of injectable contraceptive services by PMVs and the broader effects of their activities on the health system have not been investigated. Contextual factors refer to “all factors that are not part of an intervention itself” [9], in other words, they are the “key features of the environment in which the project is immersed, and which are interpreted as meaningful to the success, failure, and unexpected consequences of the interventions” [9]. We report in this paper findings from the study that identified the contextual factors that influenced provision of injectable contraceptive services by trained PMVs in Nigeria. The study was a component of a large project that assessed the processes and mechanisms of influence of training on access to and utilization of injectable contraceptive services among PMVs in Nigeria.

## Methods

### Setting

The qualitative study was conducted in three states: Cross River, Kaduna and Oyo. Face-to-face interviews were conducted in the PMVs’ shops as well as in the offices of health workers, PCN officials, and family planning (FP) coordinators where privacy was ensured. The interviews were conducted in January 2020. The study was guided by the the UK Medical Research Council’s framework for Complex Interventions. In 2000, the Medical Research Council introduced a framework which facilitated the use of appropriate methods for researchers and research support organizations and it was revised in 2006 to adjust for some limitations [10]. It has been used successfully across disciplines such as public health practice and in areas of social policy that have important health consequences, such as education, transport, and housing [10, 11].

### Participant selection

There were 27 respondents spanning NAPPMED officials, PMVs, FP coordinators, health workers, PCN officials and Program implementers. The President of NAPPMED or his designee who was involved in the previous study and knowledgeable about the intervention and outcomes was purposively selected. Two PMVs per state were randomly selected from the pool of the trained PMVs and interviewed. For health workers, two public or private health facilities that provided family planning services and were proximal (2–5 km) to the store of a selected PMV were listed. Afterward, two facilities from the list were randomly selected and health workers providing contraceptive services were interviewed. Program implementers and donor agencies who were involved with the implementation of the previous intervention were also purposively selected.

### Procedures for data collection

The interviews were conducted by the authors, assisted by trained male and female research assistants who had a minimum tertiary qualification of Ordinary National Diploma. Both the authors and research assistants had worked on previous ethnographic studies in the past and they had a 2-day training to familiarize with the study instruments. Thereafter, there were field practice sessions which also stressed the need for creating a rapport prior to engaging the respondents in the interview. NAPPMED officials were visited or informed of the study to seek their cooperation. The researchers intimated the respondents about the rationale for the survey as well as the ethical considerations at each interview.

### Data collection

The in-depth interview (IDI) and key informant interview (KII) guides for qualitative study were designed to collect qualitative information, amongst other things, on contextual factors affecting the acceptance of the intervention and delivery of services. In addition, the guide had questions focusing on community, health system, political and regulatory factors which influenced the intervention outcomes. Interviewers were trained for two days prior to commencement of data collection. Their training focused on objectives of the study, interview techniques, procedures for recruitment of study participants, data collection, and ethical issues. Prior to data collection, the tools were piloted in similar facilities not included in the study. Face-to-face interviews were conducted at the health facilities, offices and PMV shops. However, interviews with the program implementers were conducted by telephone because they were not within the study sites at the time of survey. A member of the research team served as the state team leader who provided direct daily supervision for interviewers during the period of fieldwork. Interviews were recorded on digital tape recorders and also field notes were taken during the interview sessions. The average duration of the interviews was about 45–60 min. The guide was administered in the preferred language of the PMVs: Yoruba or Hausa, and English.

### Study population

In all, 27 qualitative interviews were conducted, including seven key informant interviews (KIIs) with health care workers, four KIIs with NAPPMED state officials, three KIIs with FP coordinators from the State Ministries of Health, two KIIs with staff of the Pharmacy Council of Nigeria (PCN), four in-depth interviews (IDIs) with officials of PMVs and one KII with a program implementer (Table 1). There were 16 males and 11 females with age ranging from 27 to 64 years.

**Table 1:** Distribution of respondents for the qualitative interviews by sites

	Oyo State	Cross River State	Kaduna State	Total
Health workers	2	3	2	7
PMVs	3	4	3	10
NAPPMED Officials	1	1	2	4
Government officials	1	1	1	3
PCN officials	–	1	1	2
Donor Agency Representative	1	–	–	1
Total	8	10	9	27

### Ethical considerations

The Ethics Review Committees of the World Health Organization (WHO) and the University of Ibadan/University College Hospital, Ibadan, Nigeria approved the study prior to commencement of data collection and the assigned reference number is (UI/EC/19/0210). Informed consent was obtained from all participants, and digital recorders were used to document the interviews.

### Data analysis

Data quality measures were integrated into the data collection and transcription process. Members of the research team members listened to the audio tape and compared to the transcription to identify discrepancies. Thereafter, the transcribed data was cleaned and saved in word format. Members of the research team read the transcripts to ensure familiarization with the data, thereafter, the transcripts were coded using a coding frame developed in line with the study objectives. The coding ensured opportunities to integrate other emerging themes/sub-themes and there were series of team meetings to discuss the coding process and findings. Thereafter, we sorted the codes into categories depending on how the different codes are related and organised into themes. The qualitative interview transcripts were coded using Nvivo 12.0 software using thematic analysis. Data analysis and interpretation were done by more than one person to ensure the accuracy of the findings in terms of theme identification, understanding of concepts used and clarification of the idiomatic and metaphoric language.

### Findings

#### Contextual factors

The contextual factors identified were socio-cultural, community, government policy and regulatory framework, donors’ interest in exploring the potentials of PMVs for expanded healthcare service provision, and the failing Nigerian health system.

#### Socio-cultural factors

The culture of secrecy about the use of contraceptives and religion influenced or moderated the uptake and provision of injectable contraceptives and other family planning methods and these affected the implementation of the project. People do not have the confidence to discuss or use family planning methods for religions and cultural reasons. The quotes below underscore the socio-cultural milieu and the difficulty that PMVs face while providing injectable contraceptives in the communities:

*Like I said earlier religion and culture. There are some people that are too sensitive or how will I say it that they hold on to that practice; so it is very difficult to convince some people because I can remember a time that a man came to my shop on this family planning….. and I tried talking to him, but I saw that this man will not agree so I just let him be. PMV_Kaduna*

*Most people that are Muslims believe family planning is not useful, some Christians don’t accept it. Yesterday, someone said this thing we are doing [contraceptive service provision] is not good. He said it will get to a time the Northerners population will be more than ours… PMV_Oyo]*

*It [Family planning] is a confidential issue. Our people don’t want to be seen or heard that oh I’m on family planning. It’s like a taboo. I don’t know if you are aware in this environment. You get what I’m saying, However, since, the beginning of 2013 that general orientation is changing, otherwise before now, it’s like a taboo. Ah! It should not be heard that I’m doing family planning. It’s something I have to go and do secretly or quietly. You get what I’m saying. And on the other part, the men view it like that you are going to be loose and promiscuous [Government Regulatory Agency_ Cross River*]

### Government policy and regulatory framework for injectable service provision by PMVs

The study facilitated a favorable policy and regulatory environment for the provision of injectable contraceptives by PMVs for the duration of the study. Prior to the study, existing policy only permitted PMVs to sell a limited number of pre-packaged over-the-counter medicines. They were prohibited from selling prescription medication or conducting invasive medical procedures including selling and administering injectable contraceptives in accordance with the Essential Medicine List of the Federal Ministry of Health. If PMVs are found violating the laws, their shop may be sealed, and the drugs may be confiscated depending on the gravity of the offence. To overcome this challenge, the Federal Ministry of Health approved the provision of injectable contraceptives by PMVs during the entire project duration. As shown in the quotes below, this is an indication of political will by policy makers:

*Well, the political will was there by the state government, the regulators, Population Council and other stakeholders. So, I think everybody was willing, there was commitment. Government Regulatory Agency_Kaduna*

*I could remember last two years or three years, they [Pharmaceutical Council of Nigeria] came, insisting that our members should go and get a license, I was surprised when I saw them at the training, they went there and from that time, nothing happened again. We sat down with them, they now know that the PMVs have a very big role to play [PMV_Cross River]*

However, considering how government regulatory framework influenced the study, the expressions below revealed the perception of some participants:

*Well, I know the Pharmaceutical Council of Nigeria don’t really support this project because I remember then during the dissemination meeting, their head was part of the meeting and he spoke at length that day, that they [PMVs] are not given license to do such that ….he said a lot that day [Government Regulatory Agency_ Oyo].*

*….. and I remember that during the supervision too, the PMVs complained about being disturbed by the police force because some of them hid the safety boxes when we asked for it, some of them hid it under their table or one cupboard and why did you hide this thing, they said the police force are disturbing them and when they see it they will ask them for money…. [Government Regulatory Agency_ Oyo].*

### Failing Nigerian health system

Another contextual factor which surfaced in the implementation and outcomes of this intervention is the failing Nigerian health system. Non-availability of health-related commodities including drugs and long waiting time are some of the factors participants alluded to as contributing to the failing health system. According to a respondent, hospitals in Nigeria are “mere consultancy clinics” and PMVs have very good patronage because they are able to provide the medications and health products that clients need. The view is expressed below:

*our hospitals are mere consultancy clinics that is if you get there and you see who to consult. That’s the terrible situation we find ourselves as a people, you get it? So, that makes PMVs to receive patronage, good patronage from the community members, more than is expected. [Regulatory agency_Cross River]*

Also supporting this view is the ease that community members have in accessing injectable contraceptive services compared to health facilities as expressed below:

*They are happy because they can get it [injectables] at their reach, they can get it in their locality and their environment. Before, they only get it in the hospital and when they get to the hospital, there used to be a queue and they get discouraged but today most of our clients come here and feel confident because we take them in and they feel comfortable [PMV_Cross River]*

### Donors’ interest in PMVs and their potentials for expanded healthcare service provision

Many donors in Nigeria have identified the potential of PMVs as implementers of health care. This is because PMVs are everywhere in the community and they are already providing different types of services such as consultations, counselling and treatment to people living in the immediate communities where they operate, and referral of sick patients in hard-to-reach areas or places where there are no medically qualified healthcare providers. The quotes below illustrates this point:

*There are many projects now going on in Nigeria also trying to work with PMVs to see if they can be used not only to provide family planning service but also other primary healthcare services like ........... people are doing lots of other things with PMVs as a follow up with what we did with PMVs [*Program Implementer].

*That is why I said, after them [the Population Council Project], we had 3 partners, that came and built on what they had done, we have CHAI, NUHRI and IntergratE under Society for Family Health programme. They came and they trained other PMVs, even the health trained PMVs were trained on implants and we monitor, the state monitors them* [Government regulatory agency_Kaduna]

### Broader expected and unexpected effects of the intervention on the Nigerian health system

The intervention had expected and unexpected effects on the Nigerian health system. One of the expected effects of the project is that the PMVs bridged the Nigerian health system service delivery gaps. They are the first point of contact for injectable services in their communities. This is a positive impact of this intervention and showed that the PMVs were accepted in the communities as sources of injectable contraceptives and other healthcare services. The quote below buttressed this:

*In this community their primary point [for FP] is the vendors. …….. ….. by the time you start interacting with the patients you will find out that they have already gone to the vendors first [Health Worker_Cross River]*

Secondly, the intervention may have improved family planning service delivery with positive effect on contraceptive use in the project states. According to one of the officials of the government agency in Kaduna the intervention positively influenced the utilization of family planning in the state. The quote below underscores this:

*This study influenced the health system, we had increase in family planning service delivery; before we were not recording as much [FP service delivery] as when the PMVs came on board [Government regulatory agency_Kaduna]*

Third, the success of the intervention encouraged more partners and donors to support implementing similar interventions as the quotes below exemplify.

*there was an impact in the state after their pilot exercise, we had three partners that came in to train PMVs because of the success that was seen and realized……they now saw that they could train more of the PMVs, increase their coverage area and then train both non-health related PMVs [Government regulatory agency_Kaduna]*

*There are many projects now going on in Nigeria also trying to work with PMVs to see if they can be used not only to provide family planning service but also other primary healthcare services like ........... people are doing lots of other things with PMVs as a follow up with what we did with PMVs (*Program Implementer).

Four, the intervention facilitated more interactions between the PMVs and relevant government agencies working on health issues as expressed in the quote below:

*…….. As a result of this project we [PMVs] have been interacting with them [the State Primary Health Care Development Agency]. We have had Stakeholders’ meetings with them; more than 5 times in their secretariat …. As a result of this project, they began to interact with us, if they have any programme, they use to call us [invite us]. [PMVs _Kaduna state]*

Five, the project created multiplier effects in the sense that some trained PMVs organized step-down trainings (quote #2) to those not selected for the study.

*There is no negative impact but the only thing that I just want to say is that when we finished the training our colleagues were not happy with the fact that only few people benefitted from the training [PMV_Oyo]*

*The positive impact was that we organized a step-down training for our colleague that did not participate in the training on Sayanna injectable contraceptive because we do express love towards one another. If we see a new drug in town, we do educate ourselves on its usefulness. [PMV_Oyo]*

However, the project created two unintended effects. The first relates to the perception of the capacity of PMVs. It was found that some PMVs had exaggerated views of their right to administer injections. The concern for most of the representatives of the government regulatory bodies was that the PMVs might practise outside the scope permissible such as administration of other injections apart from injectable contraceptives.

*Not really, but on the part of the vendors like one of the monitoring I went for …..one of the vendors told us that she didn’t use to give injections before but she now knows it's her right, so all of us shouted and we had to correct her you know that it's not her right to give injections [Government Regulatory body_ Kaduna]*

Secondly, task shifting injectable contraceptive service provision to PMVs may constitute a source of interprofessional tensions and rivalry among health care workers.

*….. we have some health workers that believe that the PMVs are trying to steal their jobs from them and that is reducing the respect people have for them in the community [Government Regulatory body_ Oyo]*

## Discussion

The contextual factors which were external to the project were socio-cultural factors, government political will, increasing donors’ interest in exploring the potentials of PMVs for expanded healthcare service provision and the failing Nigerian health system. These contextual factors include the cooperation of all the partners involved in implementation of the project, and the ready accessibility of the PMV shops in communities where women live. Another critical factor responsible for the success of the study was the favorable political and regulatory environments. The inadequate formal health system which is unable to meet the health needs of the citizenry made it possible for PMVs to respond to the injectable contraceptive needs of women.

### Sociocultural factors

Concerning the socio-cultural factors that influenced women’s utilization of injectable contraceptive services provided by PMVs, findings in this study revealed that some Nigerian cultural norms view family planning as a private issue and consider it as a taboo to be discussed openly, thus, it is shrouded in secrecy [12]. The stigmatization associated with FP utilisation may limit or influence the uptake of FP services by PMVs since they are based in communities. However, since they offer confidential services and are closer to where women live and work, they may be preferred compared to conventional family planning clinics.. In addition, PMVs are known to provide a range of services including family planning and sale of other products [12].

Also, there are conflicting views as regards the uptake of family planning commodities among the major religious groups in Nigeria. Religion is a significant part of the sociocultural component of the Nigerian society with the religious leaders occupying a position of respect, influence, and authority [13]. Thus, it is suggested that advocacy efforts be targeted at religious and community leaders who are encouraged to promote contraceptive provision because of their influence [13]. Religion remains one of the most important determinant of health seeking behaviour in SSA [13]. Consequently, these religious leaders have the power to impede or enable effective uptake of contraceptive methods to support family health [13]. Thus, there is a need to identify religious leaders who have high power and interest in injectable contraceptive uptake among women, thereafter advocacy would be made to these religious leaders to ensure their buy-in and support for the initiative.

### Enabling legal and regulatory environment for contraceptive service delivery 

The important roles of PMVs in injectable service delivery is influenced largely by the legal and regulatory environment which defines their scope of practice and underpins their capacities and functions. The Nigerian government has initiated a bold step by the planned adoption of the tiered accreditation system developed by the FGN and the PCN. The PCN is a Federal Government parastatal established by an Act in 2004 to regulate and control pharmacy education, training and practice [14]. The tiered accreditation system is based on a three-tier standardized training that is based on the healthcare qualifications of the PMVs [15, 16]. However, a critical foundation for its success is the creation of an enabling environment for service delivery. This can only be achieved through an evidence-based policy reform which expands the range and quality of contraceptive services provided by PMVs. This step is critical and pivotal to other broader factors which influence their functions. The policy and regulatory reform should be encompassing and address broader systematic issues such as licensing, training of human resources in patent medicine stores, contraceptive logistics management system, data management, monitoring and supervision, as well as referral.

The provision of national policy documents contribute to the success of health programs by ensuring that service-delivery practices are understood, supported, and institutionalized throughout the health system [17]. Policies provide high-level guidance on what health services should be offered, who should provide them, where they should be provided and how they should be provided [17]. They also delineate the specific roles, responsibilities, and limitations of various levels of healthcare workers [17]. This suggests that the Nigerian government need to hasten the provision of policy documents that will ensure continuous training of PMVs and empowering the trained PMVs to administer injectable contraceptives.

### Failing health system

Health systems in Nigeria are faced with shortage of trained healthcare workers to cater for the growing population. This has led to task shifting, an approach of redistributing health tasks to available lower cadre health staff after training them. According to the WHO, “task shifting is the rational redistribution of tasks among health workforce teams. Specific tasks are moved, where appropriate, from highly qualified health workers to health workers with shorter training and fewer qualifications in order to make more efficient use of the available human resources for health”. [18] In Madagascar, community-based distribution of contraceptives by non-medically trained workers (PMVs) was a task shifting approach that extended services to traditionally underserved remote areas as developed by the policy makers within the Ministry of Health [19]. The current study showed that it is feasible to task-shift the delivery of injectable contraceptive service to PMVs. One of the reasons for the low contraceptive prevalence rate (CPR) in Nigeria is the shortage of human resource for health especially in the area of injectable service provision [20]. Task shifting injectable services to the PMVs will address the shortage of human resource for health as well as contribute to the uptake of injectable contraceptives in the country.

The health status of a family and community is influenced by the health of women who are central to the functioning of the family unit. Provision of injectable contraceptive services by PMVs will facilitate women’s access to contraception, leading to reduced unwanted pregnancies, unsafe abortions, obstetric complications and maternal death. Having adequate access to health care is a significant attribute of an effective health system and it has a direct impact on the national health indices. The location of PMVs’ shops close to where people live and work will contribute to the improvement of access to health care especially injectable contraceptives for women of reproductive age group 15–49 years. With the acceptance of PMVs providing injectable contraceptives to women in the community, they can also serve as potential community mobilisers for healthcare interventions or delivery if they are adequately trained, equipped, supervised, evaluated and properly incentivised [21]. In addition, the PMVs can also make referrals from drug shops to the primary health care facilities when services requested are not available or when there are complications, and into the community-based health insurance scheme, thereby improving universal health coverage [21].

### Donors’ interest in PMVs and their potentials for expanded healthcare service provision

Many donors have worked with PMVs in providing a range of health services due to the fact that PMV shops are found in every nook and cranny of the country [22] and are the most convenient point to access injectable contraceptives for women of reproductive age. [22–24] The range of healthcare services that is provided include: rapid diagnostic testing for malaria and administration of antimalarial like artemisinin-based combination therapy; supplements such as zinc tablets; family planning methods such as injectable contraceptives, oral contraceptives, emergency contraceptives and intrauterine contraceptive devices as well as oral rehydration salts [25]. Recently, PMVs have also been trained in the integrated community case management of childhood fever, diarrhoea and pneumonia [26]. Thus, many donor agencies see the PMVs as a way of strengthening health outcomes in the suburban and rural communities where a larger proportion of the Nigerian population reside [5, 16].

### Broader effects of the intervention on the health system

We identified some positive and negative broader effects of the intervention on the health system. Firstly, the intervention was able to bridge the injectable service delivery gaps in the formal health system because PMVs responded to the needs of women who require this service. As reported by government regulatory agent in Kaduna State, the provision of injectable contraceptive services by PMVs has improved family planning service delivery in the area. This is encouraging because Nigeria is still faced with many health challenges including low contraceptive prevalence rate (CPR) and interventions are needed to improve CPR [15, 27]. Studies have shown that front-line community health workers can improve access to- and equity of- health services [28, 29] and that essential healthcare products are core elements of patient-centered healthcare systems in places without access to formal health [30]. Even though the federal government has not formally integrated PMVs into Nigerian health system as front-line health workers, PMVs provide the first and main point of care in many communities. Thus, the unexpected effects were the unplanned expansion of the human resource for health as the PMVs were trained directly by the Population Council and the trained PMVs also passed on the training to their colleagues who were not at the training organised by Population Council. Thereafter, PMVs have been offering counselling services on family planning options, delivering injectable contraceptives and other injections safely, referring clients to the PHC offering a linkage to the formal health system, as well as practising infection prevention and control. Also, some of the PMV associations also organized refresher training programs for those trained although this was not a stated activity in the study protocol. This provided an opportunity for the trained PMVs to refresh themselves about the essentials of the Population Council’s organised training and it also served as a form of “on-the-job training” which corrected any false practice and boosted their confidence to continue administering the injectable contraceptives.

The argument is that the PMVs are not formally trained, but there are a few of them who had some training in healthcare such as retired nurses [26, 31]. and provide high quality basic health services and are consulted by the poor, rural and marginalized people who have limited access to formal health services [25, 32]. The implication is that if PMVs are trained and adequately monitored and supervised, they can increase the national CPR which will ultimately improve maternal health in the country, thus bridging the currently widened health system gaps.

Secondly, there was a healthy collaboration between different stakeholders namely the NAPPMED, NGOs, PCN, and the FMOH. This was made possible because the implementer, Population Council, supported by the funder, USAID carried all the stakeholders along all through the stages of planning, implementation and monitoring and evaluation of the project. Thus ensuring that the Population Council had the buy-in of all the partners/stakeholders from the inception. The success has motivated more NGOs to explore implementation of health interventions using the PMVs as service providers. Thirdly, the PMVs conducted step-down training for their colleagues who were not included in the initial training, thus creating multiplier effect of the intervention in the community.

Nevertheless, there are some negative broader effects on the health system. One is the perception by some PMVs that they can now operate ‘as doctors’ since members of the community have accepted them as competent service providers. This perception may lead PMVs to exceed their limit in service provision and have the potential to create tension among other categories of healthcare workers.

The confidence some of the PMVs demonstrated during interviews may account for why they did not consider it necessary for them to use the job aides. Thus, the fact that the PMVs could provide basic health services to the undeserved population should be tempered with considerations for quality assurance [25]. This study and a systematic review on the roles of PMVs in healthcare provision in Nigeria by Beyeler et al. [33] had shown that PMVs have some gaps in knowledge which imply that they still need initial, continue education and close monitoring and supervision to ensure that their service provision do not exceed their scope of practices. If the PMVs will be integrated into the government workforce in the provision of injectable contraceptives, implementation of effective training and accreditation programs for them will be required to reconcile their roles and responsibilities in provision of family planning services.

## Conclusion

This study has highlighted the contextual factors which define the roles and scope of practice of PMVs involved in injectable contraceptive service provision. These factors include sociocultural, institutional, failing Nigerian health system and strategies and interventions aimed at expanding the healthcare delivery roles of PMVs must be encompassing and address the broader contextual factors which underpins their capacities and functions.

### Recommendations

There is need for government to urgently implement the tiered PMVs accreditation system to improve the overall quality of services rendered by PMVs. Appropriate training and monitoring should be integrated into the regulation system relevant to each tier of service provider. The accreditation system should serve as the mechanism through which the PMVs are integrated into the formal health care system.

Integration of PMVs into the Nigerian Health system would facilitate links between PMVs and other healthcare workers, thus improving access to quality primary health care services.

Proper monitoring and supervision of PMVs will be required to sustain the skills acquired in training. This can be done in partnership with NAPPMED and PCN as it can be directed towards supporting continuing medical education.

The government should identify and partner with religious leaders to ensure their buy-in and support for contraceptive provision within the community.

## Data Availability

The data will be made available on request to the corresponding author.

## Abbreviations

CPR: Contraceptive prevalence rate
FMOH: Federal Ministry of Health
IDI: In-depth interviews
KII: Key informant interviews
LMICs: Low- and middle-income countries
NAPPMED: National Association of Patent and Proprietary Medicines
NAPMVs: National Association Patent Medicine Vendors
NGOs: Non-Governmental Organizations
PHC: Primary Healthcare Centre
PM: Patent medicine
PMVs: Patent medicine vendors
PPMVs: Patent and proprietary medicine vendors
PCN: Pharmacy Council of Nigeria
SMOH: State Ministry of Health
SSA: Sub-Saharan Africa
USAID: United States Agency for International Development
WHO: World Health Organization
